# Recombinant apoptosis inhibitor of macrophage protein reduces delayed graft function in a murine model of kidney transplantation

**DOI:** 10.1371/journal.pone.0249838

**Published:** 2021-04-23

**Authors:** Ji Yun Lee, Shabitha Arumugarajah, Dameng Lian, Natsumi Maehara, Aaron R. Haig, Rita S. Suri, Toru Miyazaki, Lakshman Gunaratnam

**Affiliations:** 1 Department of Microbiology and Immunology, Western University, London, Ontario, Canada; 2 Matthew Mailing Centre for Translational Transplant Studies, Lawson Health Research Institute, London, Ontario, Canada; 3 Centre for Disease Biology and Integrative Medicine, University of Tokyo, Tokyo, Japan; 4 Department of Pathology and Laboratory Medicine, Western University, London, Ontario, Canada; 5 Faculty of Medicine, Division of Nephrology, McGill University, Montreal, Quebec, Canada; 6 Division of Nephrology, Department of Medicine, Schulich School of Medicine and Dentistry, Western University, London, Ontario, Canada; National Institutes of Health, UNITED STATES

## Abstract

Reperfusion injury following cold and warm ischemia (IRI) is unavoidable during kidney transplantation and contributes to delayed graft function (DGF) and premature graft loss. Death of tubular epithelial cells (TECs) by necrosis during IRI releases pro-inflammatory mediators (e.g. HMGB1), propagating further inflammation (necroinflammation) and tissue damage. Kidney Injury Molecule-1 (KIM-1) is a phagocytic receptor upregulated on proximal TECs during acute kidney injury. We have previously shown that renal KIM-1 protects the graft against transplant associated IRI by enabling TECs to clear apoptotic and necrotic cells, and that recognition of necrotic cells by KIM-1 is augmented in the presence of the opsonin, apoptosis inhibitor of macrophages (AIM). Here, we tested whether recombinant AIM (rAIM) could be used to mitigate transplant associated IRI. We administered rAIM or vehicle control to nephrectomised B6 mice transplanted with a single B6 donor kidney. Compared to grafts in vehicle-treated recipients, grafts from rAIM-treated mice exhibited significantly less renal dysfunction, tubular cell death, tissue damage, tubular obstruction, as well as local and systemic inflammation. Both mouse and human rAIM enhanced the clearance of necrotic cells by murine and human TECs, respectively *in vitro*. These data support testing of rAIM as a potential therapeutic agent to reduce DGF following kidney transplantation.

## Introduction

Reperfusion injury following cold and warm ischemia (IRI) is an unavoidable consequence of renal transplantation. While most grafts will recover from IRI, prolonged cold and warm ischemia may lead to delayed recovery and impaired graft function, with clinically relevant consequences. Delayed graft function (DGF), defined as, the need for dialysis within 7 days of renal transplantation, affects 20–50% of deceased donor grafts (DCD) [1]. DGF not only results in acute morbidity and increased cost, but is increasingly recognized as a risk factor for rejection and long-term graft loss [2,3]. As prolonged ischemia times are often unavoidable, especially with donation after cardiac death, a better understanding of the mechanisms leading to tissue damage during IRI is needed in order to develop effective therapeutic agents.

During renal IRI, ATP depletion and reactive oxygen species production leads to apoptosis and/or, more predominantly, necrosis of the renal tubular epithelial cells (TECs) [4–7]. To add insult to injury, dying cells lose membrane integrity, releasing intracellular damage associated molecular patterns (DAMPs) into the extracellular milieu, which triggers inflammation promoting further cell death. This positive-feedback loop of inflammation and cell death–or necroinflammation–exacerbates tissue damage [8] and potentiates both allo- and non-alloimmune injury [7–9]. Although kidneys have marked regenerative capacity, such unregulated inflammation and/or severe injury can lead to irreversible allograft fibrosis [10,11]. Thus, strategies to enhance the clearance of dying cells should dampen the release of danger signals, breaking the feedback loop, and thereby protect the graft against graft damage from IRI. If this results in decreased systemic release of danger signals from the injured graft, such a strategy could mitigate augmentation of alloimmune responses [12,13].

Kidney Injury Molecule-1 (KIM-1) is an endogenous transmembrane glycoprotein transiently upregulated on proximal TECs during renal injury [14]. The upregulation of KIM-1 on renal TECs during injury transforms them into semi-professional phagocytes capable of clearing apoptotic cells (efferocytosis) [15], via direct binding of KIM-1 to phosphatidylserine on apoptotic cells. Importantly, efferocytosis by proximal TECs is solely dependent on KIM-1 [16]. We have previously reported that compared to *wild-type* mice, KIM-1 deficient or KIM-1 mutant (KIM-1 Δmucin) mice exhibited greater renal tissue damage, inflammation and mortality when their native kidneys were subjected to warm IRI [16,17]. In a subsequent study, when we exposed donor kidneys from KIM-1 deficient mice to both warm and cold ischemia and then transplanted them into *wild-type* mice. In the same study, we observed greater renal and systemic inflammation as well as increased susceptibility to renal dysfunction and tissue damage, compared to transplanted kidneys from *wild-type* donors [18]. Taken together, these studies suggest that KIM-1 plays a protective role in mitigating tissue damage by inhibiting necroinflammation [18].

While KIM-1 expression confers TECs with the capacity for direct phagocytosis of apoptotic cells, clearance of necrotic cells by KIM-1 expressing TECs is enhanced through the opsonization by the serum protein, apoptosis inhibitor of macrophage (AIM), also known as CD5L [19]. AIM is produced by macrophages to support their survival and circulates in the blood at high concentrations [20,21]. Previous studies have delineated the role of AIM in several conditions [20,22]. At steady state, AIM is bound to the much larger circulating IgM pentamer complexes, effectively preventing trafficking into the renal tissue architecture [19,21–23]. However, during acute kidney injury, AIM undergoes cleavage and dissociates from IgM pentamers through an unknown mechanism [19,21]. It is then filtered by the glomerulus and accumulates on necrotic debris within renal tubules in both humans and mice [19,21,23]. We have previously demonstrated that, during moderate warm IRI of native kidneys, filtered AIM accelerated renal recovery and improved mice survival in a KIM-1 dependent fashion [19]. However, the anti-inflammatory and therapeutic effects of rAIM in renal transplantation has not been studied.

To this end, we tested whether recombinant AIM can be used as a potential therapeutic agent to improve renal recovery after transplantation of kidneys exposed to severe warm and cold ischemia. If found to be beneficial, exogenous AIM administration could potentially be used to reduce incidence of DGF after transplantation of donor kidneys which have inevitably been exposed to prolonged ischemia, thus improving long-term graft outcomes in humans. We hypothesized that administration of exogenous recombinant AIM would augment KIM-1-mediated clearance of necrotic cells, mitigating necroinflammation, tissue damage, and renal dysfunction after transplantation of kidneys exposed to severe IRI.

## Materials and methods

### Renal transplantation/AIM administration

All mice were maintained, 4 in a cage with their litter mice with free access to food and water, ample bedding and shelters prior to transplantation. Following the transplantation procedure, each mouse was kept in its own cage, placed on top of a warming blanket, and food placed on the bottom of the cage. Environmental conditions such as the humidity and temperature were monitored and kept consistent with pre-operation conditions. Anesthesia of the animals was induced by injection with Ketamine (50-100mg/kg)/ Xylazine (5-10mg/kg) I.P. and then maintained in 2% isoflurane during the transplantation surgery. During the entire operative process, mice were kept on warming blankets (~37°C) and body temperature was measured using a rectal probe. Mice were administered with a single dose of pre-emptive buprenorphine (0.05–2.5mg/kg) at the time of anesthetic induction. A second dose of buprenorphine was given 6–12 hours later as needed. Post-operative buprenorphine injections were given twice daily for 48 hours. To provide additional supportive care, mice were administered with 0.5-1mL of 5% dextrose in saline post-transplantation. With the consultation of specialized vets, each mouse was monitored for unanticipated pain, distress or suffering by observing weight loss, loss of mobility, loss of appetite, decreased fecal/urine output, licking, vocalizing, biting, shaking, restlessness, scratching, failure to groom, abnormal resting postures or hunched up, and failure to show normal patterns of inquisitiveness. With animals being monitored twice a day for health and welfare, in case where mice experiences excess suffering or pain, or encounter weight-loss of >15%, mice were euthanized as soon as noted.

For the transplantation procedure, single kidney transplants of C57BL/6 (B6) mice kidneys into B6 recipients following bilateral nephrectomy was performed [24,25]. Donor kidneys were exposed to approximately 60 min of warm ischemia and 35 min of cold ischemia during each procedure. Following kidney transplantation, recipients were administered a single 200 μl dose of either mouse rAIM (2mg/ml), or an equal volume of PBS intravenously via the tail vein. Murine rAIM was generated as previously described and provided by Dr. Miyazaki [19,23]. All recipient mice were viable at 2 days post-transplantation at which point they were euthanized using Carbon dioxide overdose, and graft tissue and serum were collected. We chose this time point based on the literature and sustained viability of recipient mice [26]. The primary outcome was renal function at 2 days and was quantified by serum creatinine as previously described [18]. We chose this timepoint as peak graft injury and mortality occurs within the first 3 days of surgery if the graft fails [26]. We assessed tissue damage and local inflammation through histology by pro-inflammatory cytokine (IL-6, MIP-2**α**, IL-1**β**) expression in the graft, and systemic inflammation by serum HMGBI. Due to our specified early endpoint of 2 days post-transplant surgery, no mortality was observed prior to the endpoint. The number of animals used were 4-6/ group for each experiment as per the figure legends. These numbers were based on a previous study [18].

B6 mice were obtained from the Charles Rivers Laboratory. All animal procedures were pre-approved by Western University animal use subcommittee in accordance with the regulations of the Canadian Council on Animal Care.

### Histology/Immunohistochemistry

A renal pathologist, blinded to the treatment groups, scored the degree of Acute tubular necrosis (ATN) and tubular obstruction on tissue H&E sections using a previously described semi-quantitative method as follows: The ATN score was based of damage sustained to the brush border, dilation of proximal tubules, proteinaceous casts, widening of interstitial space, and necrosis (0 = 0%, 1 = <10%, 2 = 11–25%, 3 = 26–45%, 4 = 46–75%, 5 = >75%) [16]. Immunohistochemistry was performed on sections to visualize apoptotic cells using anti-cleaved Caspase-3, infiltration of neutrophils using anti-myeloperoxidase antibody, macrophages using anti-CD68 antibody, and AIM using a custom-made rabbit anti-murine AIM antibody. Spleens were used as positive control to confirm anti-cleaved Caspase-3 staining. All antibodies were obtained from Abcam, Cambridge, MA. Quantification was done by counting and averaging from 5 different fields at 400x magnification for each section. Glomeruli were excluded for the above studies. Antibodies used are as follows: Cleaved Caspase-3 (Asp175) #9661 (Cell Signaling Technology); polyclonal Anti-CD68 antibody: ab31630 (Abcam); and monoclonal, Clone number: ED1 Anti-Myeloperoxidase antibody (MPO): ab9535 (Abcam). Sections were also stained with Periodic acid-Schiff (PAS) (Sigma Aldrich, Cat#395B-1KT).

### Inflammatory markers

Tissue sections were collected in TriPure isolation reagent (Roche Diagnostic, Basel, Switzerland) and total RNA extracted. qSCRIPT cDNA SuperMix (Quanta Biosciences, Gaithersburg, MD) was used to generate cDNA. Real-time polymerase chain reaction (RT-PCR) was completed using StepOnePlus Real-Time PCR System (Applied Biosystems, Waltham, MA) and SYBR Green (Thermo Fisher Scientific, Rockford, IL) detection was used to quantify relative expression. Primers (Integrated DNA Technologies, Coralville, IA) used were: IL-6: F-5’-TACTCCTTCCTACCCCA ATTTCC-3’ R- 5’- TTGGTCCTTAGCCACTCCTTC-3’; MIP-2α: F-5’-CAAAGGCA AGGCTAACTGACC-3’ R- 5’ACATCAGGTACGATC CAGG C-3’; and IL-1β: F-5’- ACCTAGCTGTCAACGTGTGG -3’ R-5’ TCAAAGCAATGTGCTGGTGC-3’. We used GAPDH: F- 5’ TCAGCATCTCTAAGCGTGGT-3’ R-5’-ATGTTGTCTTC AGCTCGGGA-3’as an internal control.

Serum HMGB1 was quantitatively determined using Sandwich-enzyme immunoassay kit in accordance with its protocol (Shino-Test Corporation, Tokyo, Japan). Multiskan GO (Thermo Fisher Scientific, Rockford, IL) was used for quantification.

### Cell cultures

Human Embryonic Kidney 293 (HEK 293) cells and mouse kidney (Renca) cells were obtained from America Type Culture Collection (ATCC, Manassas, VA) and cultured at 37°C in 5% (vol/vol) CO_2_ incubator. HEK 293 cells stably expressing human KIM-1 pcDNA (HEK293 -pcDNA) were generated by transfecting with plasmid construct encoding human KIM-1 using Lipofectamine® 2000 (Life technologies. Thermo Fisher Scientific, Rockford, IL). Stable cell lines were maintained with geneticin (G418) sulfate (Santa Cruz Biotechnology, Santa Cruz, CA) supplemented in DMEM (Invitrogen, Carlsbad, CA) containing 10% FBS (Invitrogen, Carlsbad, CA), and 1% Penicillin-streptomycin (Invitrogen, Carlsbad, CA). Renca cells stably expressing mouse KIM-1 were generated by transducing with Lenti ORF particles, Havcr1 (Myc-DDK-tagged) or LentiORF control particles (OriGene Technologies, Rockville, MD). These stable cell lines were maintained with puromycin dihydrochloride (Sigma-Aldrich) and cultured in RPMI-1640 medium containing 10% FBS, 5% PS, 0.1mM non-essential amino acids (ThermoFisher Scientific, Waltham, MA), 1 mM sodium pyruvate (ThermoFisher Scientific), and 2 mM L-glutamine (ThermoFisher Scientific). Hallmark appearance of these cells was confirmed by visual analysis.

### Phagocytosis assay/Flow cytometry

We collected thymocytes from B6 mice and heat-killed them by incubating the cells at 65°C for 20 minutes to induce necrosis. Necrotic thymocytes were verified by flow cytometry analysis showing double positive staining for propidium iodide (Biolegend, San Diego, CA) and annexin V (Biolegend, San Diego, CA). Necrotic thymocytes were labeled with human rAIM (100 μg/ml) (R&D systems, Minneapolis, MN) at 37°C for 1 hour, or not labeled at all for control. We then performed phagocytosis assay as previously described [19]. Briefly, approximately 1x10^6^ HEK293 were plated and fed 3x10^6^ necrotic thymocytes with or without human rAIM, or no thymocytes (control). After incubation for 90 minutes at 37°C in 5% CO_2_ incubator, cells were placed on ice to reduce non-specific binding for 30 minutes, and subsequentially washed 3 times with ice-cold PBS prior to harvesting. The % phagocytosis, which represents the number of tubular cells that have phagocytosed the necrotic cells, was analyzed using BD LSR II flow cytometer (BD Biosciences, San Jose, CA). Similar procedure was performed using mouse Renca cells and mouse rAIM.

### Statistics

Continuous variables (creatinine, inflammatory markers, % phagocytosis) and tissue injury scores were compared between groups using one-way ANOVA/Student’s t- and Mann-Whitney U tests, respectively. All analyses were performed with GraphPad Prism (GraphPad Software Inc., La Jolla, CA). All data are presented as means ± SEM; p-values < 0.05 were considered statistically significant without adjustment for multiple comparisons.

## Results

### Renal function and tissue damage

**Fig 1:**
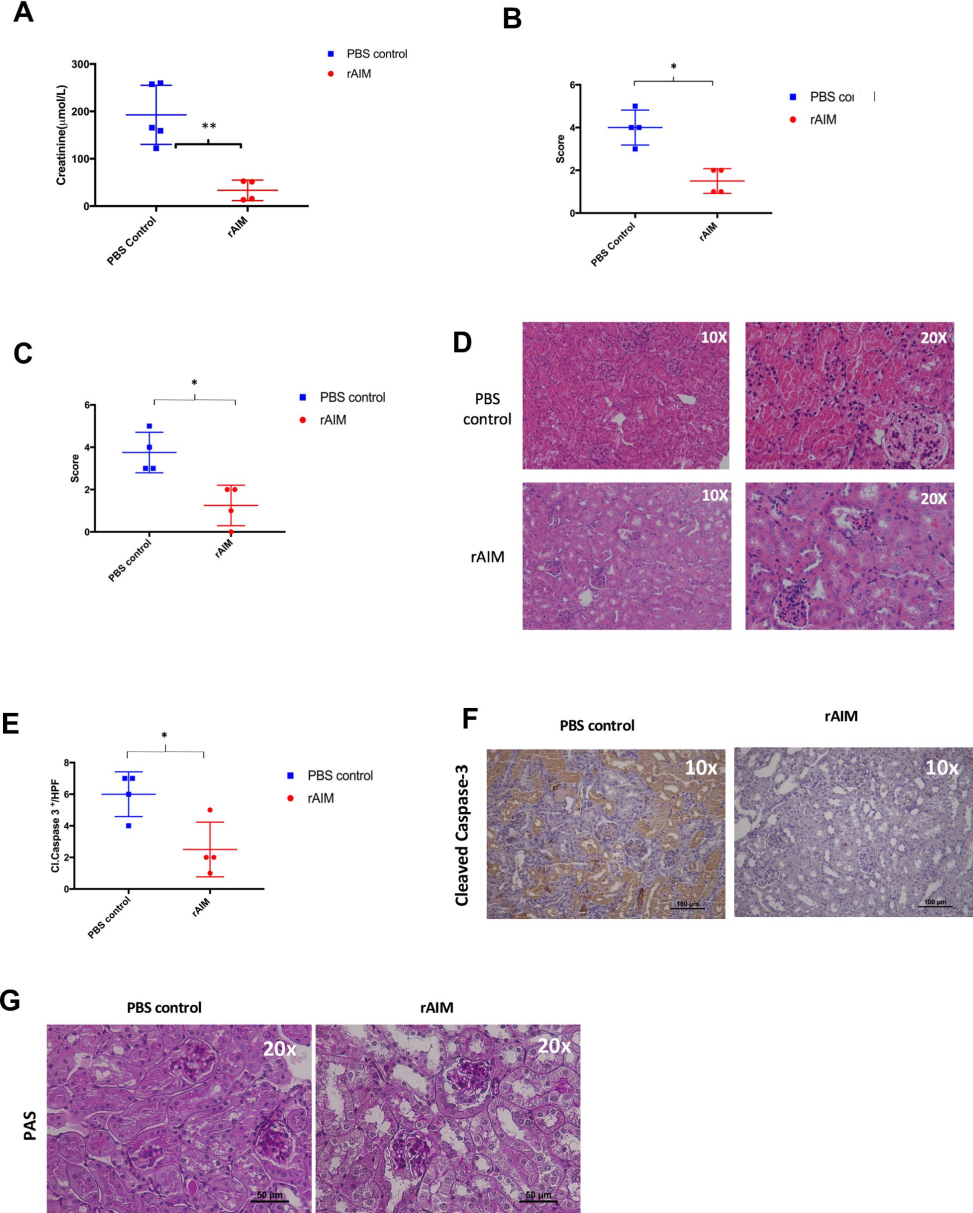
Administration of recombinant AIM improves graft function and mitigates graft damage. Donor kidneys were transplanted into B6 wild-type recipients. Following transplantation, recipients were injected intravenously (i.v.) with either 200 μl of rAIM (2mg/ml) or PBS. On day 2 post-transplantation, mice were euthanized and (A) Serum creatinine was measured as a marker of renal function (B-C) Formalin-fixed tissue sections were stained with H&E and were scored in a blinded fashion. Scoring system: 0 = none, 1 = <10%, 2 = 11–25%, 3 = 26–45%, 4 = 46–75%, and 5 = >75%. (B) ATN score, (C) tubular obstruction score (D) apoptotic tubular epithelial cells were quantified. Tubular epithelial cell staining positive staining for cleaved Caspase-3-postive were counted and averaged from 5 different fields at 400x magnification for each section (E) Images of H&E -stained tissue sections. *p<0.05, **<p<0.01, n = 4~5/group (F). Formalin-fixed tissue sections were stained for necrosis using Periodic acid-Schiff (PAS) (G).

Recipient mice who received exogenous rAIM had significantly lower serum creatinine at 2 days following renal transplantation compared to those who received PBS treated controls (33.29 ± 10.83 vs. 192.7 ± 27.86 μmol/L, p = 0.0019; Fig 1A). The rAIM treated mice also exhibited significantly less tubular necrosis (1.5 vs. 3.5, p = 0.0286; Fig 1B & 1G), less tubular obstruction (1.5 vs. 3.5, p = 0.0286; Fig 1C and 1D), and less apoptotic tubular cell death compared to PBS treated mice (2.5 ± 0.866 vs. 6 ± 0.7071, p = 0.0203; Fig 1E).

### Local and systemic inflammation

**Fig 2:**
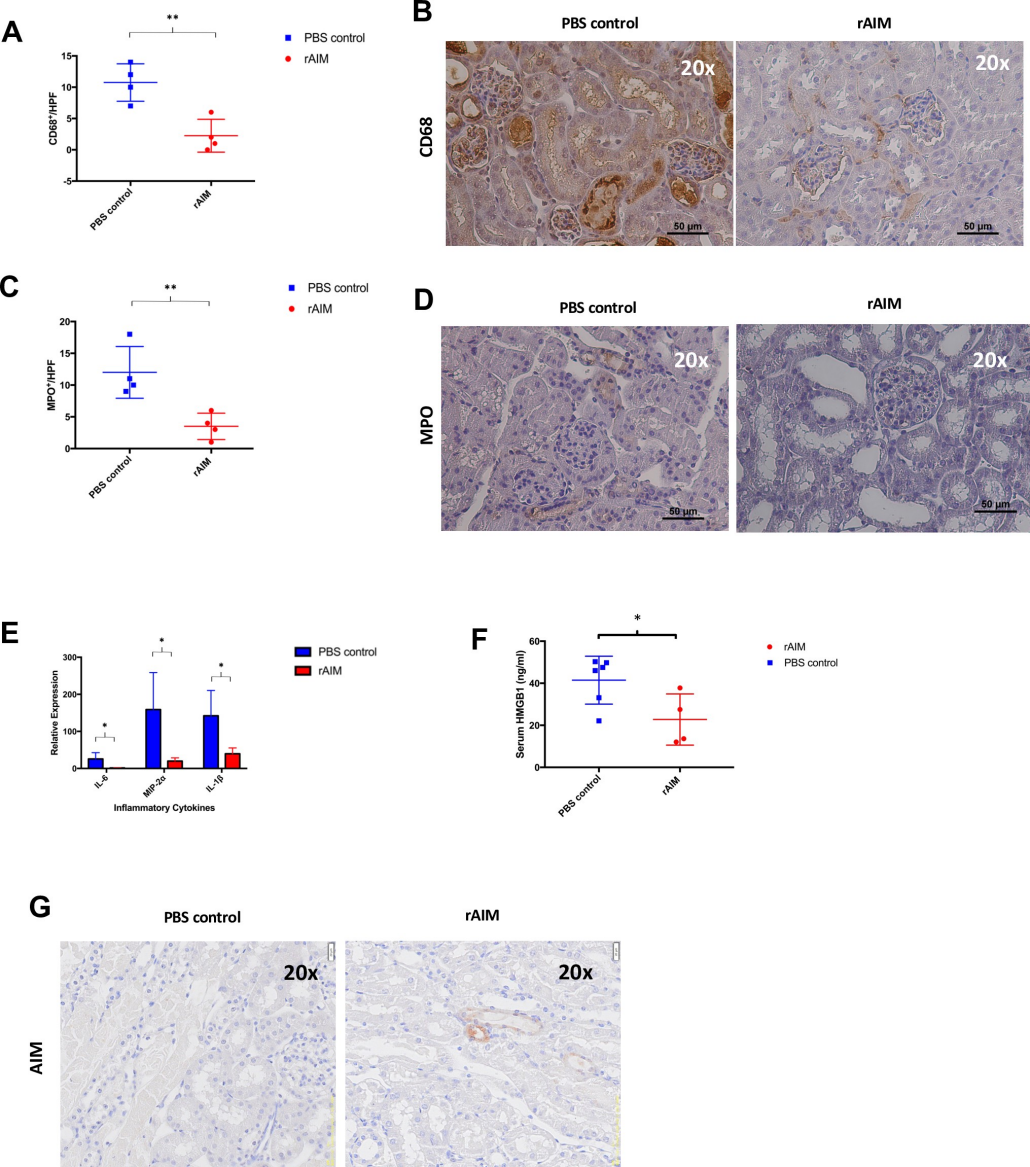
Recombinant AIM administration alleviates local tissue and systemic inflammation following syngeneic renal transplantation. (A-D) Kidney tissue sections were stained for CD68 or MPO to detect graft-infiltrating macrophages and granulocytes, respectively. (A) Number of CD68+ macrophages/HPF. (B) Immunohistochemistry images of kidney graft sections staining CD68. (C) Number of MPO+ granulocytes/HPF. (D) Immunohistochemistry images of kidney graft sections staining MPO. (E) Measurement of pro-inflammatory cytokines (IL-6, MIP-2ɑ, and IL-1β) using quantitative RT-PCR. Data were normalized to GAPDH gene expression. *p<0.05, **p<0.01, n = 4/group. (F) Serum HMGB1 levels were quantified using ELISA. p = 0.1, n = 4-6/group. (G) Kidney tissue sections were stained for AIM.

Transplanted kidneys from mice treated with rAIM exhibited significantly less macrophage infiltration (2.25 ± 1.315 vs. 10.75 ± 1.493, p = 0.0052; Fig 2A and 2B) and granulocyte infiltration (3.5 ± 1.041 vs. 12 ± 2.041, p = 0.01; Fig 2C and 2D) compared to those from PBS treated controls. Transcript analysis of transplanted kidneys revealed that grafts from rAIM treated mice had significantly less expression of pro-inflammatory genes: IL-6 (1.478 ± 0.3042 vs. 25.61 ± 8.566, p = 0.0306), MIP-2α (19.79 ± 4.45 vs. 158.8 ± 57.65, p = 0.0352), and IL-1β compared to grafts from PBS treated controls (39.71 ± 7.822 vs. 142.1 ±34.11, p = 0.0264; Fig 2E). We also observed significantly decreased serum levels of HMGB1 in rAIM-treated mice compared to PBS-treated mice (22.75 ± 6.098 vs. 40.48 ± 4.564 ng/ml, p = 0.0450; Fig 2F). Kidney grafts from mice treated with rAIM appeared to be more repaired with minimal intraluminal debris when compared to grafts from mice treated with PBS. There was also more AIM present on intraluminal debris of tubules from rAIM-treated recipients compared to PBS-treated recipients (Fig 2G) [19].

### In vitro phagocytosis

**Fig 3:**
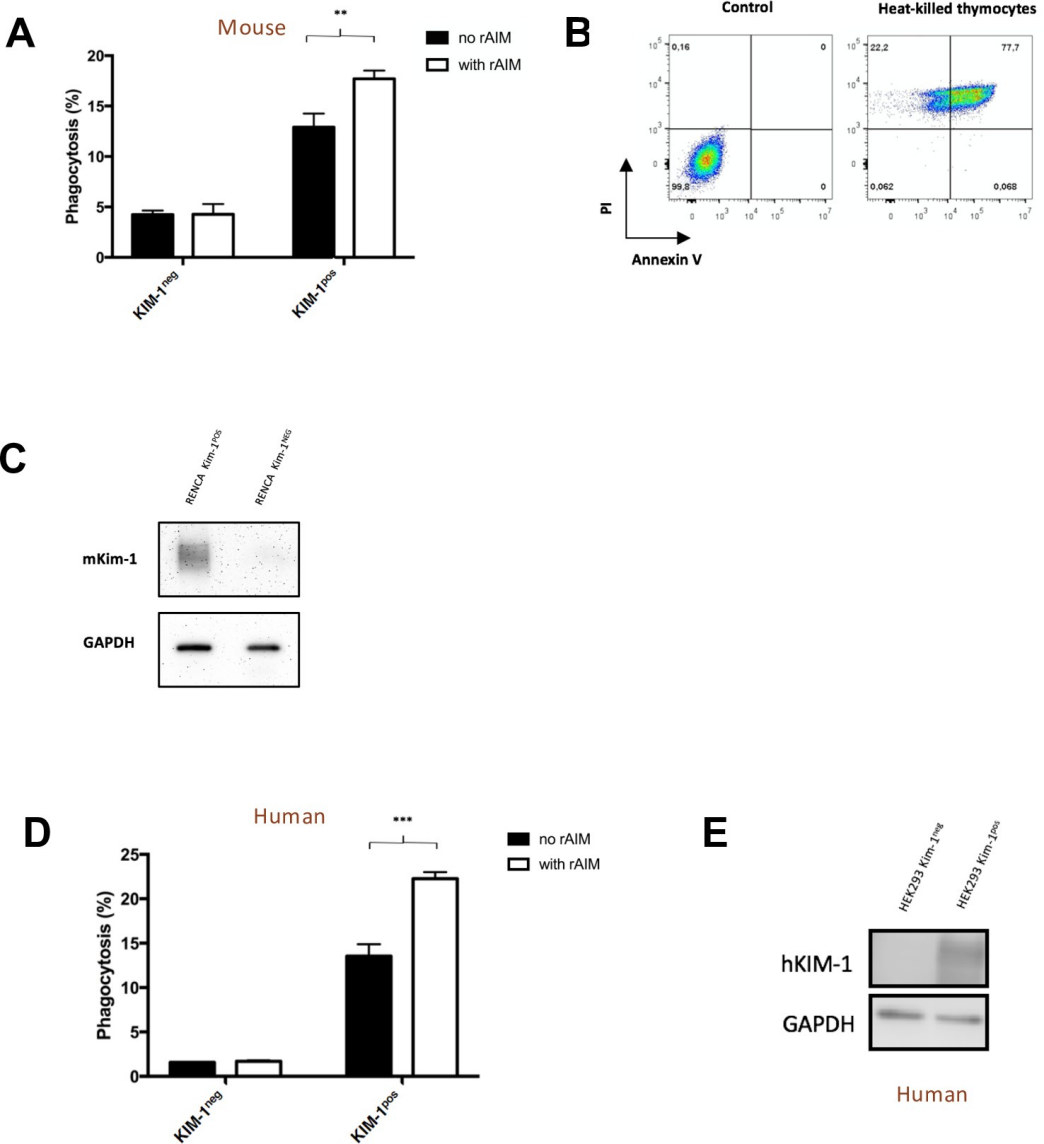
Recombinant AIM enhances the phagocytic uptake of necrotic cells by both KIM-1 expressing mouse and human kidney cells. Renca cells (mouse) or HEK293 (human) expressing KIM-1 or without KIM-1 were fed necrotic thymocytes with or without rAIM (A-C). (A) Renca cells (mouse) after a 90-minute incubation, % phagocytosis was quantified using flow cytometry. (B) Immunotyping of necrotic cell populations. (C) Western Blots were performed confirming the expression of KIM-1 on Renca cells. (D) HEK 293 cells (human) after a 90-minute incubation, % phagocytosis was quantified using flow cytometry. (E) Western Blots were performed confirming the expression of KIM-1 on HEK293 cells. Histograms are representative results of at least 3 independent experiments. All flow cytometry experiments were analyzed using FlowJo X software. **p<0.01, ***p<0.001.

Renca cells stably expressing murine KIM-1 were able to engulf necrotic cells significantly more in the presence of rAIM compared to without rAIM (17.7 ± 0.4726 vs. 12.9 ± 0.781%, p = 0.0063; Fig 3A). Phagocytosis was found to be KIM-1 dependent as Renca cells transfected with empty vector failed to clear necrotic cells. Immunophenotyping by flow cytometry confirmed that the phagocytic target cells were indeed necrotic cells, as they were either double positive for annexin V and PI or single positive for PI (Fig 3B). KIM-1 expression by the stably transfected Renca cells was verified using Western blot (Fig 3C).

Similarly, in the presence of rAIM, HEK293 cells expressing KIM-1 exhibited significantly enhanced phagocytic activity of necrotic cells compared to controls (22.27 ± 0.4333 vs. 13.53 ± 0.7753%, p = 0.0006; Fig 3D). Analogous to mouse Renca cells, rAIM-mediated phagocytosis of necrotic cells was found to be dependent on KIM-1 expression as non-KIM-1-expressing human kidney cells failed to exhibit increased clearance of necrotic cells in the presence of rAIM. KIM-1 expression on these cells was verified by Western blot (Fig 3E).

## Discussion

AIM has been reported to be of therapeutic relevance in various conditions including obesity [20], autoimmune disease [27], and hepatocellular carcinoma [22]. Here, we demonstrate the therapeutic potential of rAIM against transplant associated IRI using a clinically relevant model of kidney transplantation which incorporates severe forms of both warm and cold ischemia. We found that a single dose of rAIM administered to transplanted mice almost completely normalized renal function at 48 h post-transplant and this was accompanied by markedly reduced tissue inflammation and damage compared to PBS-treated controls. Although AIM has been shown to protect against native renal warm IRI previously [19], our study is the first to elucidate the anti-inflammatory effect of AIM in a transplant IRI setting.

As the name suggests, AIM was initially identified as an apoptosis inhibitor which ultimately supports the survival of macrophages [28]. Both humans and mice have relatively high levels of serum AIM (5μg/ml and 2μg/ml, respectively) and similar reported function [21]. With a half-life of 5 days for mice and 6 hours for humans, the majority of circulating AIM is bound to the Fc region of pentameric IgM (>500kDa), where one IgM pentamer is bound to one AIM molecule. In this state, AIM is said to be stabilized by IgM pentamers and not filtered by the glomerulus [19,28]. It is likely that the interaction between AIM and IgM-Fc is due to the positively charged domain on the AIM protein being attracted to the negatively charged area in the IgM-Fc [23]. However during kidney injury, AIM dissociates from IgM-Fc in the blood and is filtered, accumulating on necrotic debris and interacts with KIM-1 thereby enhancing the phagocytic ability of KIM-1 to clear the necrotic debris [19]. The mechanism as to how AIM dissociates from IgM-Fc is still unknown, but one speculation may be that during kidney injury, altered homeostasis may disturb the charge differences. However, this has yet to be investigated.

The main clinical manifestation of transplant IRI is DGF, resulting in increased morbidity, prolonged length of stay and increased resource utilization [1,3]. Decreasing the incidence of DGF would thus be of major benefit. This is particularly true in the current era where the use of DCD kidney donors, which is associated with the highest rates of DGF, is approaching 20% in North America [29,30]. To date, there are no effective therapies to prevent DGF. Thus, if rAIM were to be equally effective in humans as it was in mice, the therapeutic implications are likely to be of great importance.

In addition to the effect of rAIM on graft function, our data also highlighted the potent effect of rAIM administration on curtailing both graft and systemic inflammation (serum HMGB1) caused by excess necrosis in the graft. Based on our previous work [18,19], as well as data presented in Fig 2, we conclude that the anti-inflammatory effect of rAIM is mediated by the enhanced phagocytic clearance of necrotic cell debris within the injured grafts. The ability to limit DAMP release from the damaged tissue may be particularly important in the setting of allogeneic transplantation, where extracellular DAMPs have been shown to exacerbate alloimmunity [5,18,31]. Our syngeneic transplant model was well-suited to studying the effects of rAIM on transplant associated IRI, however it would be important to test whether rAIM administration will mitigate alloimmune injury to the graft, which can be persistent unlike IRI.

In addition to ameliorating inflammation, our work also demonstrated that rAIM treatment decreased dead cell debris within the tubular lumen, which also likely contributed to graft recovery given that tubular obstruction is a major mechanism contributing to a decline in the glomerular filtration rate during acute kidney injury [10]. Further, we observed that intraluminal debris within the tubular lumen was coated with AIM in the rAIM-treated mice, though most of the debris had been cleared in these mice compared to the PBS-treated mice. Surprisingly, we detected little (endogenous) AIM coating the abundant luminal debris in the PBS-treated group. Since AIM is endogenously produced by macrophages and freely filtered by the glomerulus during acute kidney injury, we expected the dead cell debris to be coated by AIM [19,21]. It is important to note that the effects if rAIM on renal recovery following IRI has only been tested in AIM null mice but not wild type (e.g. C57Bl/6) mice [19]. To our knowledge, this is the first study to investigate the role of AIM in a kidney transplant model. The relative absence of AIM in kidneys of our PBS-treated mice could be due to the unique features of the kidney transplant procedure and the absence of acute kidney in the donor mice prior to renal pedicle clamping and organ harvesting. Endogenous AIM is only excreted in the urine of mice with acute kidney injury, though the exact mechanism that links kidney injury to disassociation of AIM from IgM pentamers has not been fully elucidated [21]. Taken together with previous work by our group [16,19,21,23], our results here suggest that rAIM associates with intraluminal debris with the injured kidney grafts and enhances its rapid clearance to promote renal recovery. The relative absence of AIM in the PBS-treated transplant recipients may thus explain the dramatic beneficial effect of rAIM on graft function following syngeneic transplantation. Finally, our finding that rAIM augmented the phagocytic uptake of necrotic cells by human kidney cells expressing human KIM-1 supports the translation of our work to transplant patients.

In summary, our study is the first to delineate the therapeutic role of recombinant AIM in renal transplantation. Furthermore, our results demonstrate that KIM-1/AIM-mediated clearance of dying cells mitigates graft damage, and inflammation, while improving early graft function. Thus, administration of recombinant AIM may be used as a therapeutic strategy to improve graft outcomes in kidney transplant patients.

## Supporting information

S1 Fig Western Blots confirming KIM-1 expression. (a) Western blot confirming KIM-1 expression on Renca cells. (b) Western blot confirming KIM-1 expression on HEK293 cells (Includes KIM-1 variants).
(PDF)

S2 Fig Effect of recombinant AIM administration on serum cytokine levels following syngeneic renal transplantation. Recipient C57BL/6 mice were injected i.v. with 2 mg of rAIM or PBS following renal transplantation. On day 2 after transplantation, mice were euthanized, and serum was collected for measurement of cytokines (IFN-γ, IL-1β, IL-6, IL-17A and TNF-α) using ELISA.
(PDF)
