# Supplementary material for: Recombinant apoptosis inhibitor of macrophage protein reduces delayed graft function in a murine model of kidney transplantation
Source: PLoS One. 2021 Apr 23;16(4):e0249838. doi: 10.1371/journal.pone.0249838 (PMC8064555; doi:10.1371/journal.pone.0249838)
Supplement: S1 Fig — (a) Western blot confirming KIM-1 expression on Renca cells. (b) Western blot confirming KIM-1 expression on HEK293 cells (Includes KIM-1 variants). (PDF) [file pone.0249838.s001.pdf]

Figure 1

a)

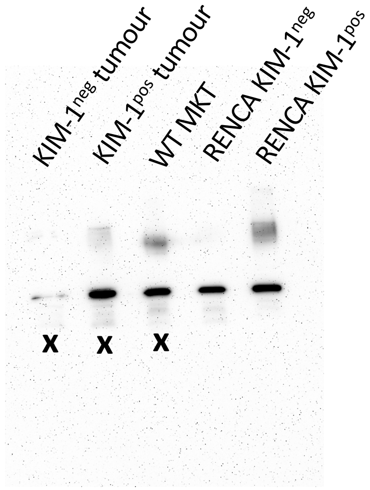

b)

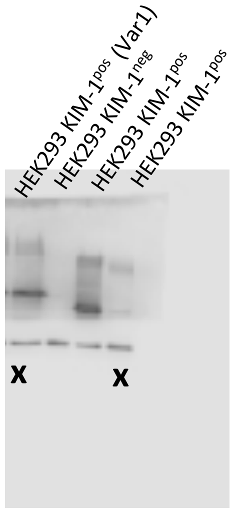

Figure 1: Western Blots confirming KIM-1 expression. (a) Western blot confirming KIM-1 expression on Renca cells. (b) Western blot confirming KIM-1 expression on HEK293 cells (Includes KIM-1 variants)
