# Supplementary material for: Recombinant apoptosis inhibitor of macrophage protein reduces delayed graft function in a murine model of kidney transplantation
Source: PLoS One. 2021 Apr 23;16(4):e0249838. doi: 10.1371/journal.pone.0249838 (PMC8064555; doi:10.1371/journal.pone.0249838)
Supplement: S2 Fig — Recipient C57BL/6 mice were injected i.v. with 2 mg of rAIM or PBS following renal transplantation. On day 2 after transplantation, mice were euthanized, and serum was collected for measurement of cytokines (IFN-γ, IL-1β, IL-6, IL-17A and TNF-α) using ELISA. (PDF) [file pone.0249838.s002.pdf]

Figure S2

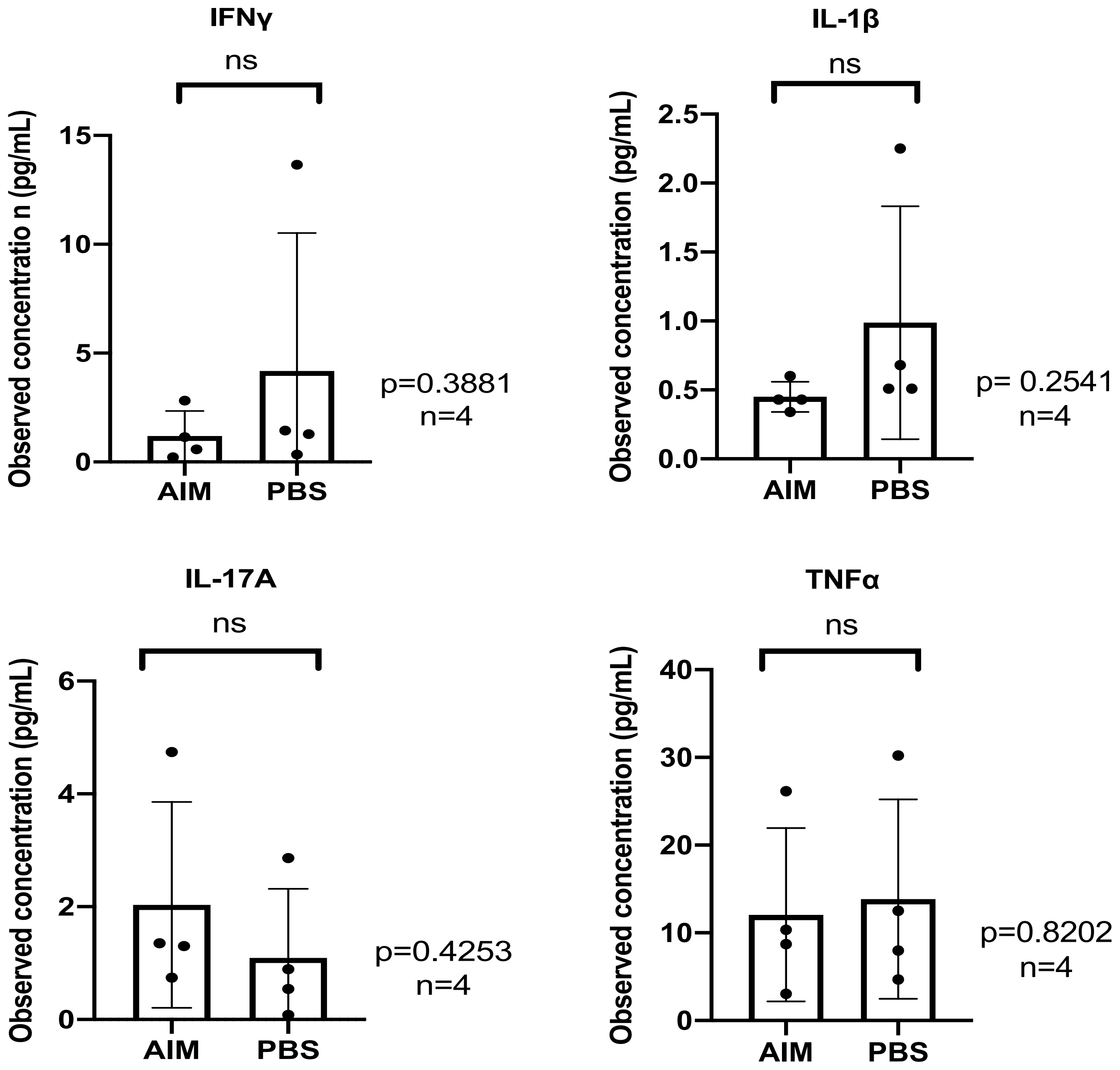

Figure S2. Effect of recombinant AIM administration on serum cytokine levels following syngeneic renal transplantation. Recipient C57BL/6 mice were injected i.v. with 2 mg of rAIM or PBS following renal transplantation. On day 2 after transplantation, mice were euthanized, and serum was collected for measurement of cytokines (IFN-γ, IL-1β, IL-6, IL-17A and TNF-α) using ELISA.
